# Contemporary Profile of Hybrid Nerve Sheath Tumors: Clinical, Anatomical, and Imaging Characteristics

**DOI:** 10.3390/cancers18183053

**Published:** 2026-09-20

**Authors:** Nadja Grübel, Anne-Kathrin Uerschels, Oliver Gembruch, Karsten Wrede, Felix Schöffing, Marc Hohenhaus, Christoph Scholz, Katharina Hobitsch, Thomas Kretschmer, Simone D’Souza, Nora F. Dengler, Christian Rainer Wirtz, Stefanie Deininger, Anja Osterloh, Juliane Bremer, Maria Teresa Pedro, on behalf of the Peripheral Nerve Tumor Study (PNTR) Study Group

**Affiliations:** 1 Peripheral Nerve Unit, Department of Neurosurgery, University of Ulm, Lindenallee 2, 89312 Günzburg, Germanymaria-teresa.pedro@uni-ulm.de (M.T.P.); 2 Department of Neurosurgery, University of Essen, Hufelandstraße 55, 45147 Essen, Germany; 3 Department of Neurosurgery, Gemeinschaftsklinikum Mittelrhein, Johannes-Müller-Straße 7, 56068 Koblenz, Germany; 4 Department of Neurosurgery, Medical Center-University of Freiburg, Faculty of Medicine, University of Freiburg, 79106 Freiburg, Germany; 5 Department of Neurosurgery & Neurorestoration, Klinikum Klagenfurt, 9020 Klagenfurt, Carinthia, Austria; 6 Epilepsy Center Frankfurt Rhine-Main, Department of Neurology, University Medical Center Frankfurt, Goethe-University Frankfurt, 60596 Frankfurt, Germany; 7 Department of Neurosurgery, Helios Hospital Bad Saarow, Pieskower Str. 33, 15526 Bad Saarow, Germany; 8 Institute of Neuropathology, University Hospital Ulm, Albert-Einstein-Allee 23, 89081 Ulm, Germany; 9 Institute of Neuropathology, University Hospital RWTH Aachen, Pauwelstraße 19, 52074 Aachen, Germany

**Keywords:** hybrid peripheral nerve sheath tumor, schwannoma, neurofibroma, perineurioma, peripheral nerve tumor surgery, neurofibromatosis, schwannomatosis

## Abstract

**Objective:** Hybrid peripheral nerve sheath tumors (HPNSTs) are rare benign neoplasms combining features of more than one peripheral nerve sheath tumor subtype—most commonly schwannoma, neurofibroma, and/or perineurioma. Multicenter registry data were used to improve their clinical, radiological, histopathological, and syndromic characterization. **Methods:** This study analyzed 55 histologically confirmed HPNSTs in 52 patients from a multicenter registry of 627 surgically treated patients at four tertiary neurosurgical centers in Germany and Austria. Demographic, anatomical, clinical, imaging, histopathological, surgical, follow-up, and syndrome-associated variables were evaluated. **Results:** HPNSTs were identified in 52/627 surgically treated registry patients (8.3%), comprising 55 lesions. Median age at surgery was 57 years (IQR 44–63 years; range, 18–78), and 27/52 patients (51.9%) were female. Lower-extremity or lumbosacral lesions were more common than upper-extremity or cervicothoracic lesions (32 vs. 23 cases). Schwannoma/neurofibroma hybrid was the predominant subtype (51/55, 92.7%), followed by schwannoma/perineurioma and triphasic schwannoma/neurofibroma/perineurioma tumors (2/55 each, 3.6%). All tumors were benign. Provoked pain was the leading symptom (47/55, 85.5%), whereas motor deficits were uncommon (5/55, 9.1%). MRI frequently showed inhomogeneous contrast enhancement (42/48, 87.5%) and cystic components (19/47, 40.4%). Complete resection was achieved in 49/55 tumors (89.1%). A documented or suspected syndromic association was present in 18/52 patients (34.6%). **Conclusions:** In this surgically selected multicenter registry cohort, HPNSTs accounted for 8.3% of operated peripheral nerve tumor patients. This cohort frequency should not be interpreted as population prevalence. The integrated clinical, imaging, pathological, and surgical data highlight pain burden, multifocality, and potential syndromic associations. Long-term behavior cannot be determined because follow-up was short and incomplete.

## Simple Summary

Hybrid peripheral nerve sheath tumors are rare benign tumors that contain features of more than one type of nerve sheath tumor. Because most published reports focus mainly on pathology, little is known about how these tumors present clinically, where they occur, how they appear on imaging, and how they behave after surgery. In this multicenter registry study, we analyzed 55 hybrid nerve sheath tumors in 52 patients treated at four specialized neurosurgical centers in Germany and Austria. Most tumors combined features of schwannoma and neurofibroma, commonly caused pain, and were frequently located in the lower extremities. Complete surgical removal was achieved in most cases. No malignant transformation was documented during the available, predominantly short follow-up. A relevant proportion of patients had a documented or suspected underlying tumor-predisposition syndrome. These findings improve the clinical characterization of surgically treated hybrid nerve sheath tumors; however, they do not estimate population prevalence or long-term recurrence risk.

## 1. Introduction

Hybrid peripheral nerve sheath tumors (HPNSTs) are rare, predominantly benign peripheral nerve sheath neoplasms characterized by the coexistence of histomorphological and immunophenotypic features of at least two conventional nerve sheath tumor entities, most commonly schwannoma, neurofibroma, and/or perineurioma [1,2]. Although described in the pathology literature for decades, HPNSTs were formally recognized within the World Health Organization (WHO) classification framework in the context of soft tissue tumors in 2013 and subsequently incorporated into later tumor classifications [3,4,5]. The designation encompasses morphologically distinct combinations rather than a single uniform tumor type.

Histopathological diagnosis requires the identification of distinct or intermingled components showing differentiation toward more than one conventional peripheral nerve sheath tumor lineage. Schwannian areas typically express S100 and SRY-box transcription factor 10 (SOX10), whereas perineurial differentiation may be supported by epithelial membrane antigen (EMA), glucose transporter 1 (GLUT1), or claudin-1 expression [2,6,7,8]. However, the relative proportions of the individual components can vary substantially, and limited biopsy material may not adequately represent the heterogeneous architecture of the lesion. Distinction from conventional schwannoma, neurofibroma, perineurioma, and malignant peripheral nerve sheath tumor may therefore be challenging, particularly in tumors with focal atypia, elevated proliferative activity, or incomplete immunohistochemical characterization.

Available evidence suggests that HPNSTs occur predominantly in young adults, show no clear gender predilection, and generally follow a benign clinical course, although robust long-term outcome data are scarce [6,7]. Schwannoma/perineurioma hybrids predominate in pathology-centered series and are often sporadic [6,7], whereas hybrid neurofibroma/schwannoma tumors (HNS) are particularly relevant in the setting of neurocutaneous tumor-predisposition syndromes, including neurofibromatosis type 1 and schwannomatosis (SWN)-spectrum disorders [7,9,10]. Following the 2022 international consensus update, SWN is now used as an umbrella term that includes neurofibromatosis type 2 (NF2)-related, SMARCB1-related, LZTR1-related, and not otherwise classified forms [11]. Accurate recognition of HNS is clinically important because misclassification as pure neurofibroma may erroneously suggest neurofibromatosis type 1 (NF1), while the underlying condition may lie within the SWN spectrum [12]. Recent molecular studies indicate subtype-specific heterogeneity: recurrent erb-b2 receptor tyrosine kinase 2 (ERBB2) mutations have been identified in a subset of HNS, particularly in sporadic SWN, whereas recurrent vestigial-like family member 3 (VGLL3) rearrangements characterize many schwannoma/perineurioma hybrids and related fusion-driven benign nerve sheath tumors [13,14,15,16]. These observations support interpreting hybrid morphology in the context of histological subtype and syndromic background, but they do not permit molecular status to be inferred in untested tumors. Occasional local recurrences and rare malignant transformations have been reported; their true incidence remains uncertain [6,17].

The clinical relevance of recognizing a hybrid phenotype extends beyond pathological classification. Preoperative magnetic resonance imaging (MRI) findings are generally nonspecific and may resemble those of conventional benign peripheral nerve sheath tumors, limiting reliable prediction of hybrid histology before surgery [9]. Nevertheless, awareness of a possible hybrid tumor may support representative tissue sampling and careful clinicopathological correlation. When these tumors arise from major peripheral nerves, surgical management must balance tumor removal against preservation of neurological function. Furthermore, tumor multiplicity or a HNS phenotype may prompt evaluation for an underlying schwannomatosis-spectrum disorder and consideration of genetic counseling [10,11,12].

Most published data on HPNSTs derive from pathology series and case reports [6], frequently centered on cutaneous or soft tissue lesions and even less reported with spinal involvement [18]. A dedicated radiological series provided MRI–pathology correlation in 24 tumors [9], but integrated information on symptoms, carrier-nerve anatomy, imaging, operative strategy, neurological outcome, and longitudinal follow-up remains limited. Comparisons between published series are further complicated by heterogeneous inclusion criteria, variable pathological work-up, and differing referral and selection patterns [6,7,8]. Consequently, the clinical phenotype of HPNSTs encountered in specialized peripheral nerve surgery cannot be derived reliably from pathology-centered literature alone.

Consequently, clinically orientated multicenter data may provide complementary insights into HPNSTs encountered in routine peripheral nerve surgery, while remaining subject to referral and surgical selection bias. The objective of this study was to use data from the multicenter Peripheral Nerve Tumor Registry (PNTR) in Germany and Austria to establish a contemporary clinical, anatomical, radiological, histopathological, and surgical profile of histopathologically confirmed HPNSTs. We analyzed demographic and anatomical patterns, histopathological subtypes, clinical and neurological presentation, MRI characteristics, extent of resection, early follow-up, tumor multiplicity, and associations with tumor-predisposition syndromes, including SWN-spectrum disorders. The principal contribution is an integrated, multicenter perspective on nerve tumor surgery rather than an estimate of population prevalence or a molecular characterization of HPNSTs. 

## 2. Materials and Methods

### 2.1. Study Design and Setting

This registry-based multicenter observational study is nested within the German PNTR and is registered with the German Clinical Trials Registry (www.drks.de, accessed on 18 September 2026) [19]. The analysis included patients treated at four tertiary neurosurgical centers in Germany and Austria: The Peripheral Nerve Unit at BKH Günzburg at Ulm University (35 patients and 38 lesions), University Hospital Essen (*n* = 13), University Hospital Freiburg (*n* = 3), and Klagenfurt, Austria (*n* = 1). The source registry comprised 627 surgically treated registry patients; the study cohort included 55 histopathologically confirmed HPNST lesions in 52 patients.

Patients included in the registry between 2016 and July 2026 were screened for this study. The analysis was restricted to peripheral, extradural, extracranial, and extraspinal lesions; intracranial, cutaneous, and non-nerve-associated soft-tissue lesions were not part of the PNTR sampling frame. Only patients with a histopathologically confirmed diagnosis of an HPNST were included. Diagnoses were established by the neuropathology departments of the participating centers from the combined histomorphological and immunophenotypic findings available in routine care. Neither a centralized neuropathology re-review nor a prespecified uniform immunohistochemical panel was performed. In diagnostically uncertain cases, the responsible local neuropathologist considered external reference pathology.

Captured variables included patient age and sex, tumor side, anatomical compartment, upper- versus lower-extremity localization, presumed nerve of origin, histological subtype, Ki-67 proliferation marker (Ki-67)/MIB-1 antibody (MIB-1) proliferation marker/immunohistochemical proliferation index where available, pre- and postoperative pain and neurological status, maximum tumor diameter on MRI, contrast-enhancement pattern, cystic components, extent of resection, perioperative complications, follow-up status, tumor multiplicity, and syndromic associations. The registry did not prospectively record a preoperative radiological suspicion of hybrid histology or systematically score proposed signs such as a double-rim pattern or asymmetric enhancement. Accordingly, the imaging comparison was exploratory and cannot establish a diagnostic MRI signature for HPNST. A documented syndromic association was defined as a diagnosis recorded in the source medical record, based on clinical and/or molecular evaluations available in routine care. A suspected association denoted a registry-based clinical suspicion without a documented definitive diagnosis, based principally on tumor multiplicity and the available history. Germline testing and the molecular evidence supporting each recorded diagnosis were not predefined registry variables and were not systematically available; therefore, the number of formally tested patients cannot be reported reliably.

### 2.2. Statistical Analysis

Descriptive statistics were used to summarize cohort characteristics. Continuous variables were reported as medians with interquartile ranges (IQRs) and ranges; categorical variables were reported as counts and percentages using available-case denominators. Missing values were not imputed. Exploratory comparisons with conventional schwannomas were performed because schwannoma is the principal preoperative differential diagnosis in peripheral nerve surgery. The SWN-spectrum size subgroup was examined descriptively to explore whether syndromic association explained the observed size distribution. Continuous variables were compared using the Mann–Whitney U test, and categorical variables were compared using Fisher’s exact test. A two-sided *p*-value < 0.05 was considered statistically significant. Given the small subtype groups, incomplete follow-up, and very low number of outcome events, no multivariable predictor analysis was considered statistically reliable. Statistical analyses were performed using GraphPad Prism 11 (GraphPad Software, Boston, MA, USA).

## 3. Results

### 3.1. Cohort Characteristics

Among 627 surgically treated registry patients, 55 HPNST lesions in 52 patients were identified, corresponding to a cohort frequency of 8.3% among operated registry patients. The median age at surgery was 57 years (IQR 44–63 years; range, 18–78), with a slight female predominance at the patient level (27/52, 51.9%) and lesion level (29/55, 52.7%). Tumors involved the lower extremity or lumbosacral region in 32 cases (58.2%) and the upper extremity or cervicothoracic region in 23 cases (41.8%). Deep lesions were slightly more common than superficial lesions (31/55, 56.4% vs. 24/55, 43.6%), and laterality was nearly balanced (27 left, 49.1%; 28 right, 50.9%).

### 3.2. Anatomical Distribution

The most frequent carrier nerves were the sciatic nerve (*n* = 10), brachial plexus (*n* = 7), ulnar nerve, peroneal nerve, lumbosacral plexus (*n* = 6 each), tibial nerve (*n* = 5), median nerve (*n* = 4), medial antebrachial cutaneous nerve (*n* = 3), sural and vagus nerves (*n* = 2 each). Single lesions involved the saphenous nerve, intercostal nerve, femoral nerve, and digit III (Table 1).

### 3.3. Histopathology

**Figure 1:**
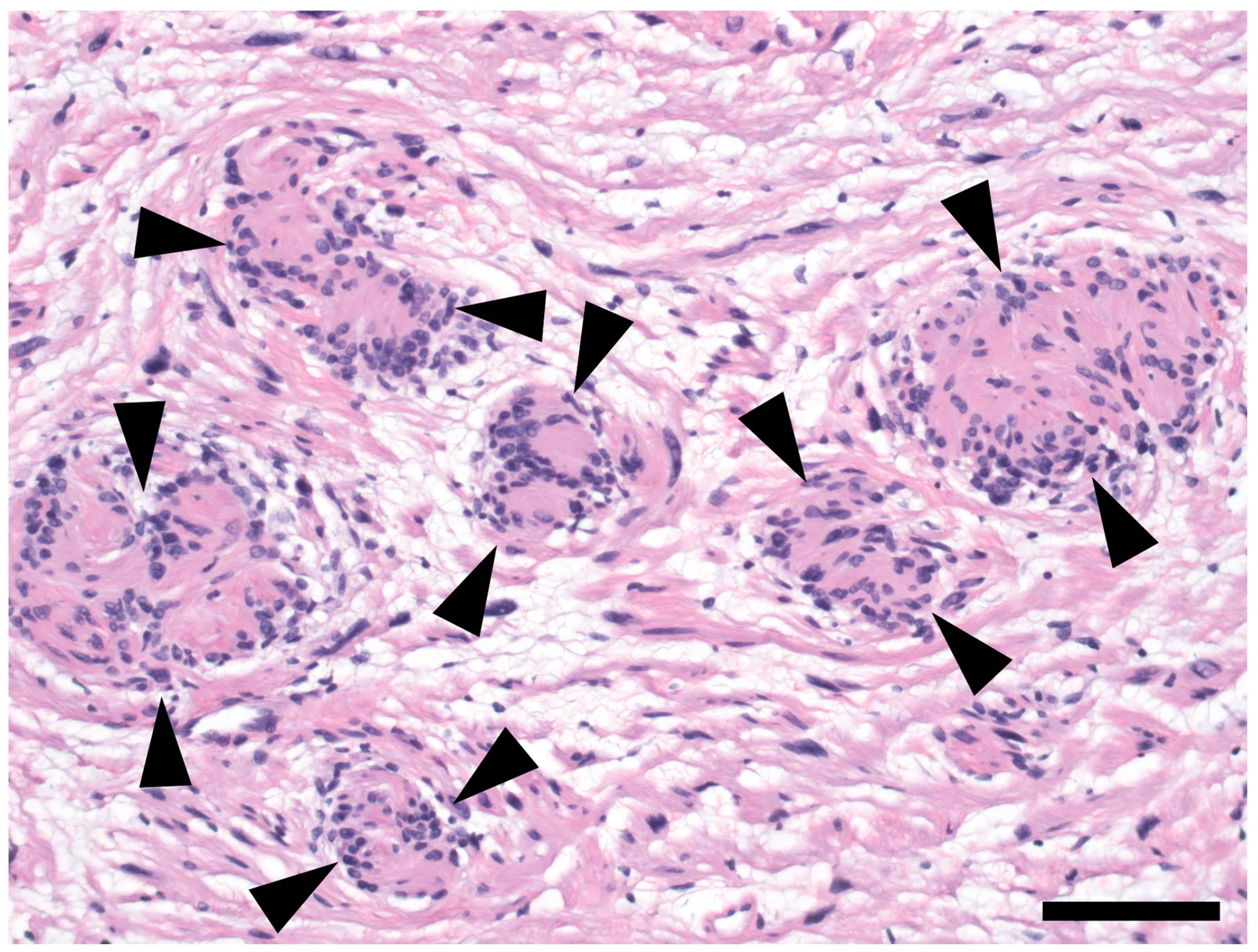
Hybrid peripheral nerve sheath tumor composed of neurofibroma and schwannoma. Loosely textured neurofibromatous tumor with typical collagen fibers, reminiscent of “shredded carrots” with characteristic spindle-cellular schwannoma islands (between arrowheads); scale bar = 100 µm.

The dominant histological subtype was hybrid neurofibroma/schwannoma (HNS; 51/55, 92.7%). Schwannoma/perineurioma was identified in two tumors (3.6%), and two tumors showed triphasic schwannoma/neurofibroma/perineurioma morphology (3.6%) (Table 2, Figure 1). Ki-67/MIB-1 information was available for 41/55 tumors (74.5%): 22/41 (53.7%) had a proliferation index <3%, 14/41 (34.1%) had a proliferation index of 4–6%, and 5/41 (12.2%) had a proliferation index >6%. All tumors in the >6% category had an index of 10%. One triphasic hybrid tumor showed an overall low Ki-67/MIB-1 index, with focal hotspot areas reaching approximately 10–15%.

Immunohistochemical data were incompletely available because staining panels were determined by local neuropathological practice and were not standardized across centers. S100 staining was documented in 41/55 tumors (74.5%), with strong or retained expression in all evaluable cases. Cluster of differentiation 34 (CD34) results were available in 37/55 (67.3%) and predominantly highlighted spindle-cell networks in hypocellular, fibrous, or neurofibroma-like areas. EMA was evaluable in 33/55 tumors (60.0%), showing capsular positivity in 19/33 (57.6%) and negative staining in 14/33 (42.4%). Reference neuropathological review was explicitly documented for five tumors. These histopathological, proliferative, immunohistochemical, and reference pathology findings are summarized in Table 2. Figure 1 illustrates the characteristic histological appearance of a hybrid peripheral nerve sheath tumor composed of neurofibroma and schwannoma. The neurofibromatous component shows a loosely textured architecture with characteristic collagen fibers resembling “shredded carrots,” interspersed with distinct spindle-cellular schwannoma islands.

### 3.4. Imaging Characteristics

Maximum tumor diameter was available for 51 HPNST lesions, and data from 263 conventional schwannomas in the PNTR were extracted for comparison. HPNSTs were larger than schwannomas, with a median diameter of 30 mm (IQR, 21–50 mm; range, 9–170 mm) versus 25 mm (IQR, 17–36.5 mm; range, 4–118 mm) for schwannomas (Mann–Whitney U test, *p* = 0.0234). Within the documented or suspected SWN-spectrum subgroup, maximum tumor diameter was available for 18 lesions with a median of 25.0 mm (IQR, 16.75–46.0 mm; range, 9–170 mm). After the four suspected SWN lesions were excluded, 15 lesions with a documented SWN-spectrum association remained for size analysis; their median maximum diameter was 24.0 mm (IQR, 13.0–51.0 mm; range, 9–170 mm). “Documented” does not necessarily indicate molecular confirmation because the underlying testing evidence was not systematically captured.

**Figure 2:**
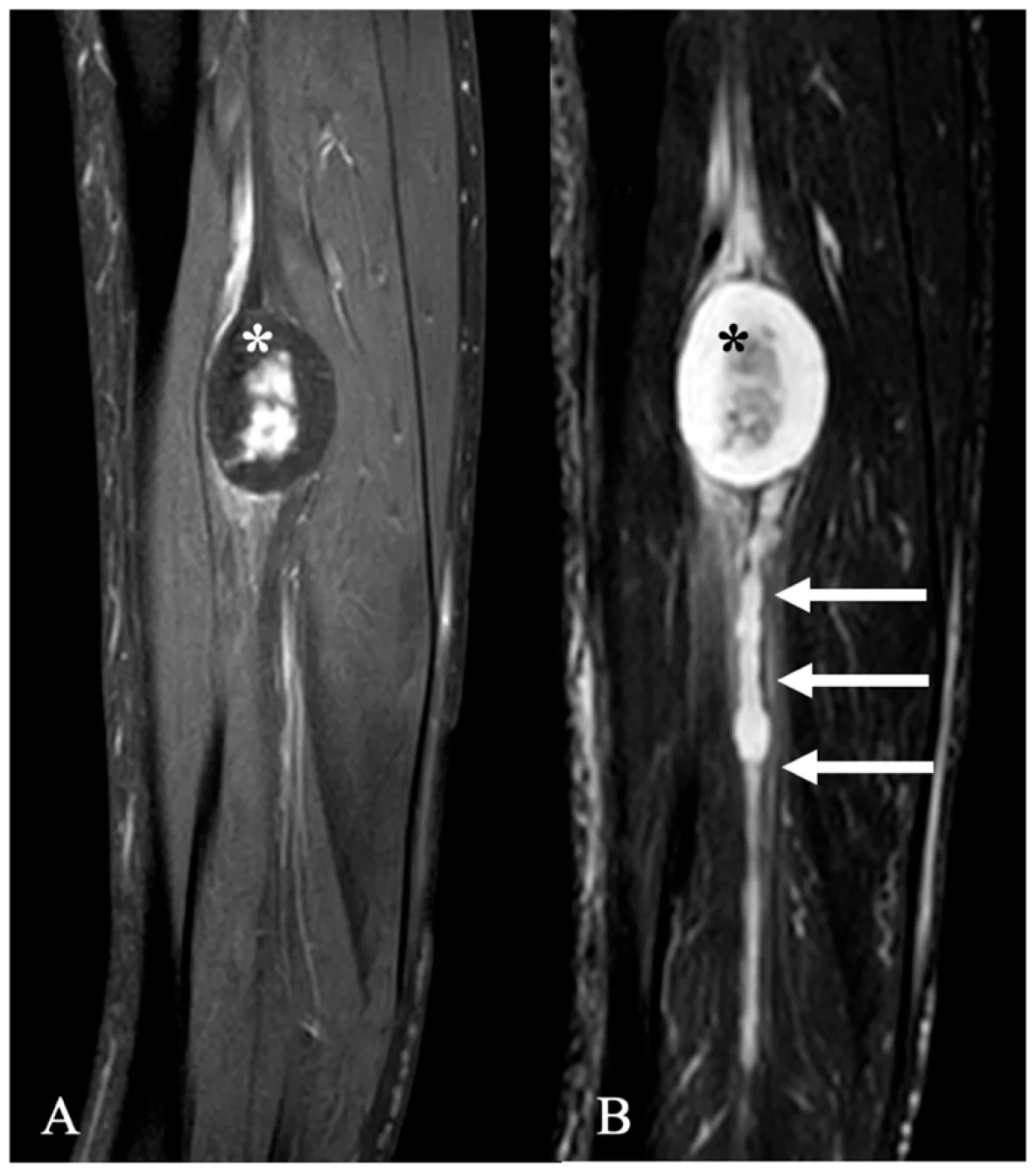
Histopathologically confirmed triphasic HPNST of the tibial nerve. Sagittal MRI of the lower leg demonstrates an ovoid, well-circumscribed peripheral nerve sheath tumor arising from the tibial nerve (*). (**A**) Contrast-enhanced fat-suppressed T1-weighted image showing inhomogeneous contrast enhancement. (**B**) Sagittal STIR sequence showing a predominantly hyperintense lesion. The tumor marked by asterisks shows a schwannoma-like appearance. The arrows indicate a longitudinally extending nerve-associated component distal to the main tumor mass, which may radiologically correspond to the perineurioma or neurofibroma component of this triphasic hybrid tumor.

**Figure 3:**
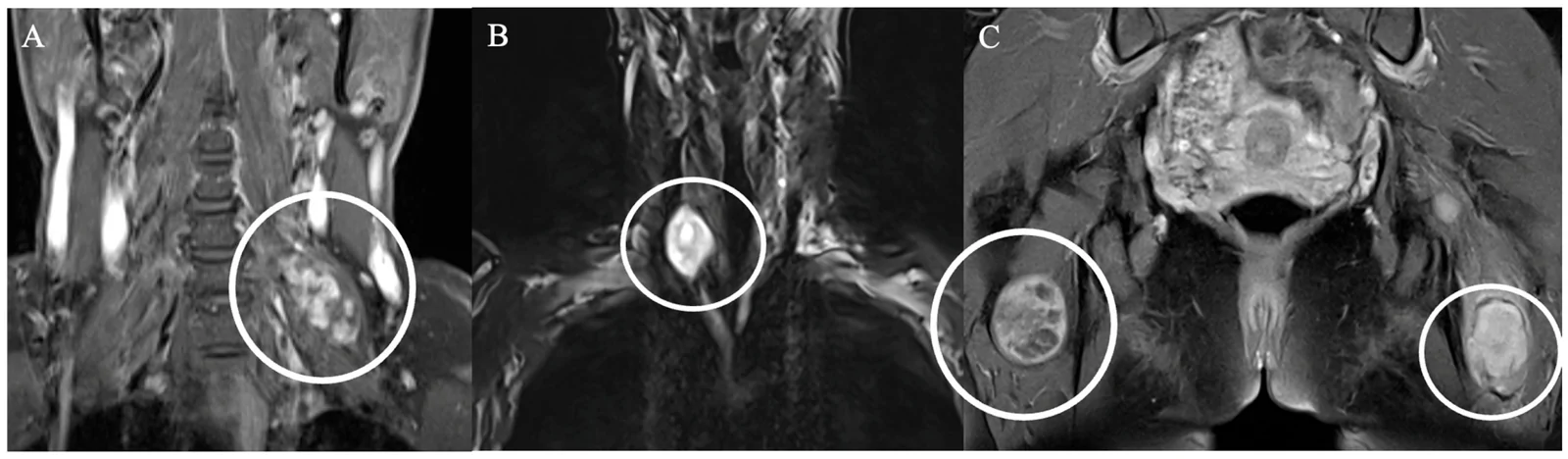
Representative MRI findings of four hybrid peripheral nerve sheath tumors (HPNSTs). Coronal contrast-enhanced, fat-suppressed T1-weighted MR images demonstrating representative hybrid schwannoma/neurofibromas at different anatomical sites. (**A**) Heterogeneously enhancing, well-demarcated lesion of the brachial plexus. (**B**) Ovoid, well-circumscribed enhancing lesion arising from the right vagus nerve. (**C**) Bilateral inhomogen contrast-enhancing lesions of the sciatic nerves. All four tumors were histologically diagnosed as hybrid schwannoma/neurofibromas. Lesions are highlighted by white circles.

Inhomogeneous contrast enhancement was more frequent in HPNSTs than in schwannomas, occurring in 42/48 evaluable HPNST lesions (87.5%) compared with 129/239 evaluable schwannomas (54.0%; Fisher’s exact test, *p* < 0.001). Cystic changes were observed in 19/47 evaluable HPNST lesions (40.4%) and 54/189 evaluable schwannomas (28.6%); although numerically more common in HPNSTs, this difference did not reach statistical significance (Fisher’s exact test, *p* = 0.158). Imaging data were missing for contrast-enhancement pattern in 7/55 HPNST lesions and 24/263 schwannomas and for cystic changes in 8/55 HPNST lesions and 74/263 schwannomas, most commonly because of incomplete preoperative MRI data. Representative MRI findings of HPNST are visualized in Figure 2 and Figure 3.

### 3.5. Clinical Presentation

**Figure 4:**
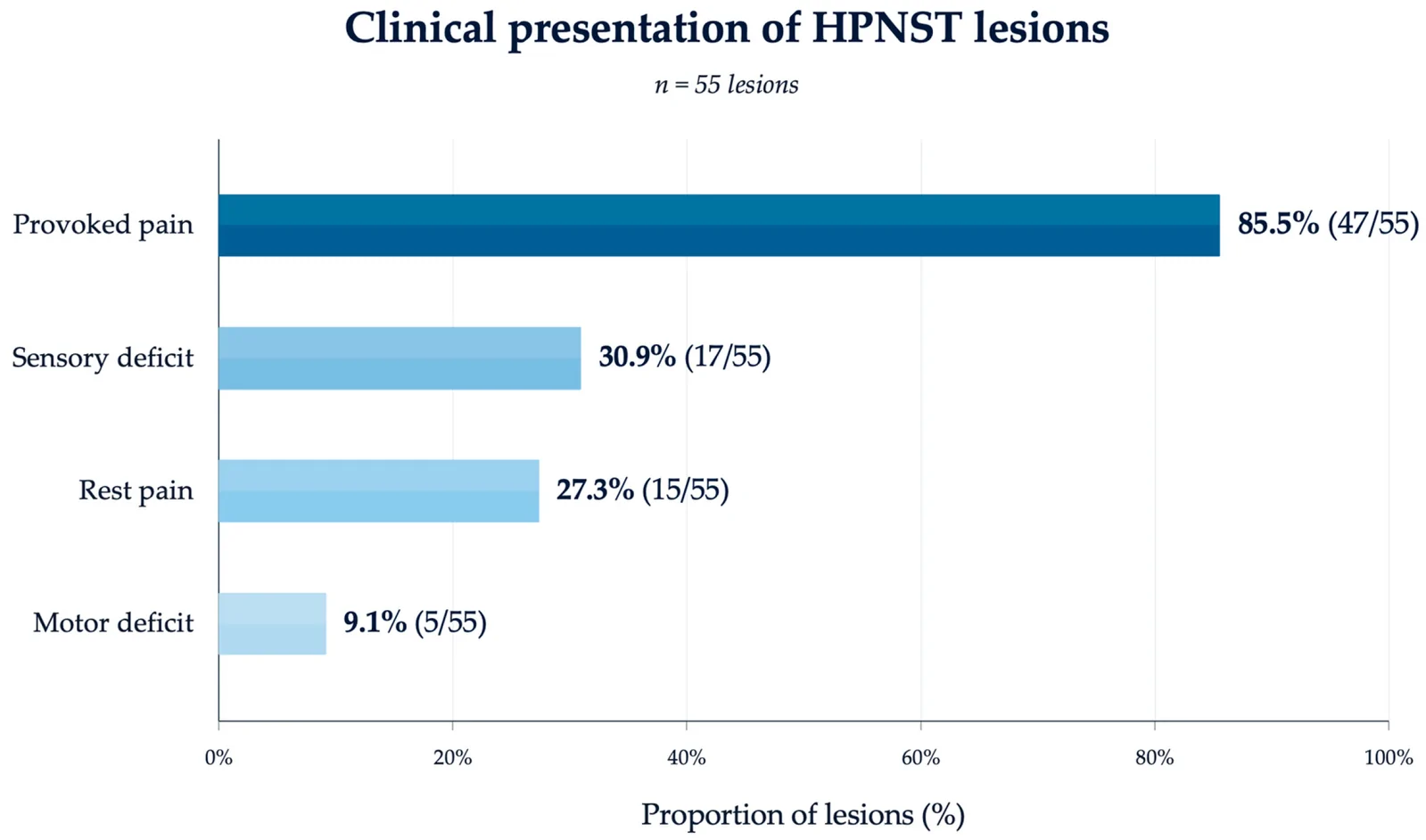
Preoperative clinical presentation of hybrid peripheral nerve sheath tumors (HPNSTs). Provoked pain was the most common preoperative symptom (47/55 lesions, 85.5%), followed by sensory deficit (17/55, 30.9%), rest pain (15/55, 27.3%), and motor deficit (5/55, 9.1%).

Pain was the predominant clinical symptom. Preoperatively, exertional, provoked, or pressure-related pain was reported in 47/55 lesions (85.5%), whereas rest pain was present in 15/55 (27.3%), as shown in Figure 4. During postoperative follow-up, persistent pain was documented in 10 cases. Preoperative sensory deficits were documented in 17/55 lesions (30.9%). Any postoperative sensory deficit was recorded in 24 lesions; 14 were coded as permanent and 9 as transient, with one lesion coded in both categories and two postoperative sensory deficits without definitive temporal classification. Motor deficits were uncommon preoperatively (5/55, 9.1%). Any postoperative motor deficit was documented in eight lesions; two were coded as permanent and four as transient, while temporal classification was incomplete for the remaining cases.

### 3.6. Surgical Treatment and Outcomes

Complete resection was achieved in 49 cases (89.1%), and partial resection in 6 cases (10.9%). Partial resection was undertaken in both triphasic hybrid tumors, which involved the vagus nerve and tibial nerves (Figure 2). In addition, partial resection was performed in two retroperitoneal tumors arising from the lumbosacral plexus and in two tumors arising from the brachial plexus. Immediate postoperative assessment was available for all 55 lesions. When these assessments were coded as 0 months, the median follow-up was 3 months (range, 0–42 months). Follow-up beyond the immediate postoperative period was available for 39/55 lesions (70.9%), with a median of 6 months (range, 1–42 months); 16/55 lesions had only an immediate postoperative assessment. Within the available follow-up, one recurrent tumor, one possible small residual lesion, and one progressive residual lesion were recorded. These observations are descriptive only and do not permit estimation of recurrence risk or durable tumor control. One postoperative hemorrhage was treated conservatively, and no wound-healing complication was documented.

Fifteen patients did not return for an assessment beyond the immediate postoperative period, either because surgery was recent and follow-up was pending or because appointments were canceled in the absence of symptoms. Because some patients contributed more than one lesion, this patient-level count is not identical to the 16 lesions without follow-up beyond 0 months.

### 3.7. Syndromic Association and Multiplicity

**Figure 5:**
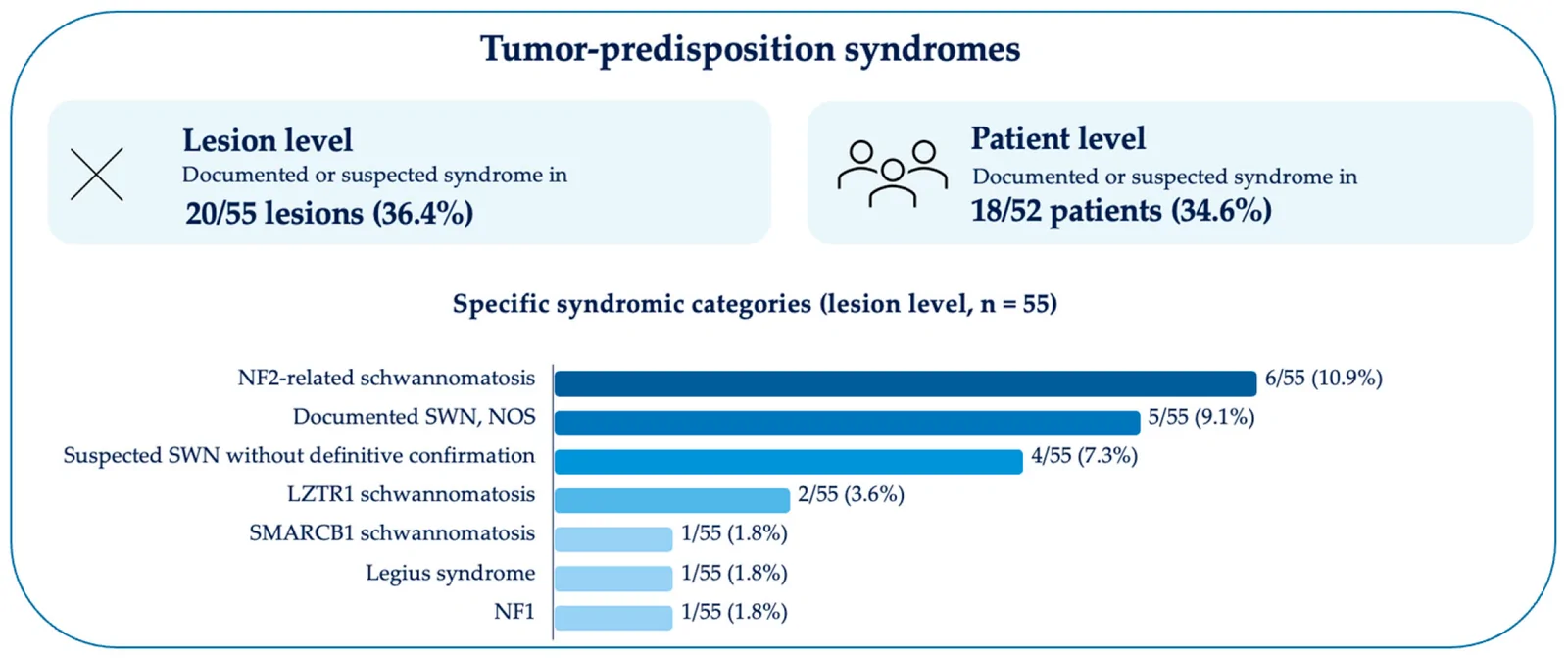
Tumor-predisposition syndromes in patients with hybrid peripheral nerve sheath tumors. Documented or suspected syndromic associations were present in 20/55 lesions (36.4%), corresponding to 18/52 patients (34.6%). The lower panel shows the distribution of specific syndromic categories at the lesion level, with NF2-related schwannomatosis being the most frequent. Note: SWN = schwannomatosis; NOS = not otherwise specified; NF1 = neurofibromatosis type 1.

A documented or suspected tumor-predisposition syndrome was present in 20/55 lesions (36.4%), corresponding to 18 of 52 patients (34.6%). Documented associations comprised NF2-related SWN (*n* = 6 lesions), SWN not otherwise specified (*n* = 5), LZTR1-related SWN (*n* = 2), SMARCB1-related SWN (*n* = 1), Legius syndrome (*n* = 1), and NF1 (*n* = 1); suspected SWN without definitive confirmation accounted for four lesions (Figure 5). The registry did not systematically capture whether each documented diagnosis was based on formal germline testing, clinical criteria, tumor testing, or a combination thereof. Consequently, the number of the 18 patients who underwent germline testing cannot be reconstructed reliably.

Overall, multiple nerve tumors were documented or resected in 12 patients, more than two tumors in 7 patients, and more than five tumors in 3 patients. All patients in this subgroup had a documented or suspected tumor-predisposition syndrome. The remaining tumors were histologically classified as conventional schwannomas, except for two additional lesions, which were HPNSTs and therefore included in the analysis. Because some patients had also undergone resections at external institutions, a complete reconstruction of all previous operations and the total number of schwannomas was not possible. This is a common limitation in this patient group, in whom the surgical history may be complex and difficult to reconstruct retrospectively.

Interestingly, two sisters with genetically confirmed SWN each underwent resection of two HNS tumors. One had bilateral mirror-image sciatic nerve tumors resected for pain, whereas the other had HNS tumors of the sciatic and vagus nerves.

## 4. Discussion

### 4.1. Key Findings and Clinical Relevance

This multicenter registry-based analysis integrates clinical presentation, nerve-level anatomy, MRI features, histopathological subtype, surgical extent, early neurological outcome, and syndromic context for 55 surgically treated HPNST lesions in 52 patients. Its principal contribution is not a mechanistic or population-based estimate, but a comparatively large real-world profile from specialized peripheral nerve surgery and an exploratory direct imaging comparison with conventional schwannomas. HPNSTs accounted for 8.3% of operated registry patients; this cohort frequency supports diagnostic awareness but cannot be extrapolated to the general population or to cutaneous and non-nerve-associated soft-tissue lesions [20,21,22,23,24,25,26]. HPNSTs in this cohort showed a predominance of lower-extremity locations and a slight female predominance at the lesion level, with the sciatic nerve emerging as the most frequent carrier nerve. Second, most lesions were HNS, in contrast to the existing literature, which reports schwannoma/perineurioma hybrids more frequently [6,9]. Some studies report a higher prevalence among young adults and no significant gender predilection [6]. Our cohort predominantly comprised middle-aged adults with a median age at surgery of 57 years. A female predominance has also been noted by Singh et al. and Lenartowicz et al. [8,9].

For context, Hornick et al. reported 42 hybrid schwannoma/perineuriomas, Singh et al. correlated MRI and pathology in 24 HPNSTs, and a 2025 review summarized 112 published hybrid tumors across heterogeneous reports [6,7,9]. A recent large single-center series by Simpson et al. identified 20 hybrid schwannoma/perineuriomas among 531 patients with solitary schwannomas, corresponding to 3.8% of the cohort. This proportion was lower than the 8.3% observed in our registry among surgically treated patients with peripheral nerve tumors who had HPNSTs. Simpson et al. included only solitary schwannomas and excluded patients with neurofibromatosis, schwannomatosis, or multiple tumors suggestive of genetic predisposition. Their hybrid tumors were exclusively schwannoma/perineuriomas, whereas HNS predominated in our cohort. These differences likely reflect cohort composition, inclusion criteria, and referral patterns, highlighting the strong influence of clinical setting on reported HPNST frequency and subtype distribution [27].

The present series is distinguished by its multicenter nerve tumor surgery setting and integrated clinical and operative variables, while the differing sampling frames preclude direct epidemiological comparison.

### 4.2. Clinical Features and Their Implications

The clinical presentation in our cohort was dominated by pain rather than objective neurological deficits, with provoked or pressure-related pain occurring in 47/55 cases (85.5%), whereas motor impairment was uncommon (5/55, 9.1%). This pattern is broadly consistent with reports on conventional benign PNSTs, in which pain, Tinel-like provocation, paresthesia, and sensory disturbance are common presenting features, while relevant motor deficits are less frequent and are more often associated with tumors involving major motor nerves [28,29].

### 4.3. Surgical Strategy, Extent of Resection, and Postoperative Outcome

Complete resection was achieved in 89.1% of lesions, whereas partial resections were concentrated in plexus, vagus, and other anatomically complex tumors where functional preservation may outweigh complete removal. Early postoperative morbidity mainly involved sensory disturbances; however, severity and duration were not captured in a standardized manner. Follow-up beyond the immediate postoperative period was available for only 39 lesions and had a median duration of 6 months. The recorded residual, progressive, and recurrent events therefore provide only a short-term descriptive snapshot and neither confirm a low recurrence rate nor establish durable tumor control. No malignant transformation was documented during the available follow-up, but the study was not designed or sufficiently powered to estimate transformation risk.

Particularly in the context of an underlying tumor-predisposition syndrome, nerve-sparing surgery remains clinically relevant, as it can provide substantial symptomatic benefit, particularly pain relief, while emphasizing the need for careful patient selection and functional preservation in patients with multiple or complex tumors [30].

Notably, the very low local recurrence rate reported in the literature [6] was largely supported by our cohort, although the limited follow-up duration restricts definitive conclusions regarding durable tumor control.

### 4.4. Anatomical Location

A predominance of lower-extremity tumors has previously been reported by Hornick et al. [7], and this pattern was also observed in our cohort. However, several anatomical findings in our series differ from much of the published literature. HPNSTs have frequently been described as dermal, subdermal, or soft-tissue-associated lesions, often involving the fingers or superficial subcutis [2,6,7,31,32]. This distribution was not confirmed in our data: none of the tumors were classified as cutaneous, although one superficial upper-extremity lesion was located at the third digit. Similarly, head and neck locations have been reported more frequently in previous series [7,9], whereas our cohort showed a clear predominance of lower-extremity and deep nerve-associated lesions.

This discrepancy likely reflects case selection and referral patterns. The PNTR captures surgically treated, usually symptomatic nerve-associated tumors from specialized referral centers and excludes conventional cutaneous and non-nerve-associated soft tissue lesions. Superficial dermal or subcutaneous lesions are often managed outside specialized neurosurgical peripheral nerve units, whereas the PNTR predominantly captures symptomatic nerve-associated tumors that require microsurgical treatment. Deep lesions and tumors affecting major nerves may therefore be overrepresented, while small asymptomatic and superficially managed lesions are underrepresented. The subtype distribution and cohort frequency should consequently be interpreted as characteristics of this selected surgical population, not as epidemiology of all HPNSTs.

Two tumors in our cohort involved the vagus nerve. Although the vagus nerve is formally a cranial nerve, both lesions were located along its extracranial cervical course and therefore fulfilled the inclusion criteria for peripheral, extracranial nerve tumors. Reports of HPNSTs arising along cranial nerves are rare [33], largely limited to individual case reports, and may be underrepresented in published series, particularly those involving the trigeminal and vestibulocochlear nerves [34]. Nevertheless, these cases highlight that HPNSTs may occur along cranial nerves and reinforce the importance of considering syndromic associations, particularly in patients with multiple nerve sheath tumors or tumors involving both cranial and peripheral nerves.

### 4.5. Imaging Phenotype and Diagnostic Pitfalls

Based on routine MRI findings, HPNSTs may be difficult to distinguish from conventional benign peripheral nerve sheath tumors, particularly schwannomas. Singh et al. reported that HPNSTs rarely demonstrate classical MRI signs of benign peripheral nerve sheath tumors, such as the target sign, fascicular sign, entering or exiting nerve sign, and split-fat sign, whereas lobulated morphology and ancient-type or cystic changes were more commonly observed [9]. In our cohort, target and split-fat signs were not specifically evaluated, as all included lesions were preoperatively considered peripheral nerve-associated tumors, and these features were therefore not expected to reliably distinguish hybrid tumors from other benign tumors. However, in line with the observations by Singh et al. [9], subtle differences were noted, particularly the relatively frequent occurrence of cystic components in 19/47 evaluable lesions (40.4%) and inhomogeneous contrast enhancement in 42/48 evaluable lesions (87.5%).

Overall, MRI identified nerve-associated lesions but did not reliably distinguish HPNSTs from conventional schwannoma. The inhomogeneous enhancement and cystic changes observed in this cohort are non-specific. The double-rim and asymmetric appearance illustrated in Figure 1 was not prospectively scored and should be regarded as an illustrative observation rather than a validated diagnostic sign. Definitive diagnosis remains dependent on histopathological and immunohistochemical assessment.

### 4.6. Histopathology and Subtype Distribution

A notable finding of this surgically selected registry cohort was the predominance of HNS. A 2025 review reported hybrid schwannoma/perineurioma in 76% of published cases (85/112), HNS in about 20% (22/112), hybrid perineurioma–neurofibroma in about 3% (3/112), and triphasic tumors were exceedingly rare (2 cases) [6]. Other pathology-centered series similarly emphasized schwannoma–perineurioma hybrids [7,8,9,32]. In contrast, 51/55 tumors (92.7%) in the present cohort were HNS, with only two schwannoma–perineurioma hybrids and two triphasic tumors. This difference may reflect awareness and referral patterns, restriction to surgically treated nerve-associated lesions, and exclusion of cutaneous and soft-tissue tumors. It should not be interpreted as evidence that HNS predominates in the population. Nevertheless, the finding indicates that HNS is clinically relevant in peripheral nerve surgery and may be underrecognized when heterogeneous tumors are sampled or classified as pure schwannoma or neurofibroma [12].

No hybrid perineurioma/neurofibroma was identified in our cohort. These tumors have been reported in association with NF1, and rare cases of malignant transformation have been described. Because these tumors are rare, their classification remains unsettled, and it is unclear whether they constitute a truly distinct tumor entity or represent neurofibromas with a prominent perineurial cell component [6,8,23].

The hybrid concept is supported by combined histomorphological and immunohistochemical features [1,2,6,7]. Schwann-cell-associated markers such as S100 and SOX10, together with perineurial markers such as epithelial membrane antigen, claudin-1, or GLUT1, where relevant, help delineate the respective components [2,6,7,8].

Molecular findings increasingly suggest subtype-specific heterogeneity rather than a single molecularly uniform HPNST entity. ERBB2-mutant neurofibroma/schwannoma hybrids may form a distinct methylation subgroup [16], whereas recurrent VGLL3 fusions support a molecularly coherent subset of schwannoma/perineurioma and related fusion-driven benign nerve sheath tumors [13,14,15]. Earlier methylation studies nevertheless placed some HNS close to schwannomas [27,28]. Marker panels and molecular testing were not standardized or quantitatively recorded in the PNTR, and no central pathology re-review was undertaken; the present study therefore cannot test these molecular associations or compare immunophenotypic profiles across subtypes. Accurate distinction from pure neurofibroma remains clinically important because misclassification may suggest NF1 when the underlying disorder is within the SWN spectrum [12]. Although HPNSTs are now an established distinct tumor entity and DNA-methylation profiling allows for the differentiation of many tumors of the nervous system, Capper et al. [35] have so far failed to demonstrate a clear distinction from schwannoma [36]. This may either suggest that molecular differences do not manifest at the epigenetic DNA-methylation level, or alternatively that they may represent a morphological variant rather than a separate molecular entity. Regardless of this ongoing classification debate, accurate distinction from pure neurofibroma is clinically important, as misclassification may lead to an erroneous diagnosis of NF1, whereas the underlying condition may be SWN, including NF2-, LZTR1-, or SMARCB1-associated disease [12].

In selected tumors, proliferative activity varied between sampled areas, including focal increases in Ki-67/MIB-1 labeling. This observation supports representative sampling but does not establish biological aggressiveness. The small number of tumors with higher indices, sparse outcome events, and short follow-up precluded a meaningful association analysis between Ki-67/MIB-1 and recurrence or progression.

### 4.7. Syndromic Association and Tumor Multiplicity

HPNSTs have been reported in association with tumor predisposition syndromes—particularly NF2-related and other forms of SWN, and less often NF1 [6,10,37]. In this cohort, a documented or suspected association was recorded for 20/55 lesions and 18/52 patients; all patients with multiple resections belonged to this subgroup. These findings require caution because germline testing was not systematic and evidence supporting recorded diagnoses was incompletely captured. The registry also did not systematically record whether an earlier NF1 diagnosis had subsequently been revised to SWN after recognition of HNS. One patient remained recorded as having NF1, but diagnostic reclassification cannot be evaluated from the available variables. Malignant transformation appears to be rare overall in both NF1-associated and non-NF1-associated HPNSTs, although NF1-associated tumors may carry a comparatively higher risk of progression to MPNST; precise rates remain unclear and are primarily based on a few case reports [6,38]. No malignant transformation was documented during the available follow-up; no inference about transformation risk can be made from this study.

## 5. Limitations

This study has several limitations. First, follow-up was short and incomplete: only 39/55 lesions had assessment beyond the immediate postoperative period, with a median of 6 months, precluding reliable estimates of recurrence, malignant transformation, durable tumor control, or long-term neurological recovery. Second, the PNTR is a surgically selected referral-center cohort enriched for symptomatic, deep, and major-nerve lesions; cutaneous, non-nerve-associated soft-tissue, conservatively managed, and asymptomatic tumors are underrepresented or absent. The observed 8.3% is therefore a cohort frequency, not population prevalence. Third, histopathological evaluation was decentralized, with no central re-review, uniform immunohistochemical panel, or quantitative marker-level registry data. Fourth, germline and tumor-molecular testing was not systematic, and the basis of each documented syndromic diagnosis could not always be reconstructed; clinically suspected associations must not be equated with molecular confirmation. Fifth, postoperative deficits were not graded with a standardized severity scale, symptom duration was incompletely recorded, and a single primary surgical indication was not captured. Finally, available-case analysis, missing MRI and Ki-67 data, small subtype groups, and very few outcome events limited subgroup and predictor analyses. These constraints define the study as descriptive and hypothesis-generating.

## 6. Strength

Key strengths of this study include its multicenter design, the comparatively large cohort size relative to previously published series of hybrid peripheral nerve sheath tumors, and the availability of detailed clinical data alongside robust histopathological confirmation. This combination enables a clinically meaningful characterization of this rare tumor entity beyond purely pathology-based descriptions.

## 7. Conclusions

Within this multicenter surgical registry, HPNSTs accounted for 8.3% of operated peripheral nerve tumor patients, comprising 55 lesions in 52 patients. To our knowledge, this represents one of the largest clinically characterized multicenter cohorts of HPNSTs reported to date and provides a comprehensive contemporary profile integrating clinical presentation, nerve-level anatomy, imaging, histopathology, surgical management, early outcomes, and syndromic context. HNS was the predominant subtype in this nerve-surgery cohort, contrasting with the predominance of schwannoma/perineurioma in pathology-centered series and highlighting the influence of clinical setting and referral patterns on reported subtype distributions. The findings emphasize pain as the leading clinical manifestation, the frequent involvement of lower-extremity and deep peripheral nerves, and the importance of considering an underlying tumor-predisposition syndrome. Complete resection was achieved in most cases, supporting the feasibility of effective surgical management in specialized centers. Although the observed frequency should not be interpreted as population prevalence, this large multicenter cohort substantially extends the available clinical evidence on these rare tumors and provides a relevant basis for future prospective studies incorporating standardized pathology review, molecular characterization, functional outcome assessment, and longer follow-up.

## Figures and Tables

**Table 1:** Baseline cohort characteristics and anatomical distribution of HPNSTs. Among 627 surgically treated registry patients, 52 had histologically confirmed HPNSTs, corresponding to a cohort frequency of 8.3% among operated patients. Three patients each had two HPNST lesions, yielding 55 tumors. At both the patient and lesion levels, a slight female predominance was observed. Most tumors were deeply located and more frequently involved the lower extremity or lumbosacral region. The sciatic nerve was the most common carrier nerve.

Variable	Value, *n*/N (%)
Cohort level characteristics	
Source registry, patients	627
Patients with HPNST	52/627 (8.3%)
Female	27/52 (51.9%)
Male	25/52 (48.1%)
Age at surgery, median (range), years	57 (18–78)
Lesion level characteristics	
Total HPNST lesions	55
Lesions in female patients	29/55 (52.7%)
Lesions in male patients	26/55 (47.3%)
Deep localization	31/55 (56.4%)
Superficial localization	24/55 (43.6%)
Left-sided lesions	27/55 (49.1%)
Right-sided lesions	28/55 (50.9%)
Lower extremity/lumbosacral locations	32/55 (58.2%)
Upper extremity/cervicothoracic locations	23/55 (41.8%)
Nerve or anatomical site	
Sciatic nerve	10/55 (18.2%)
Brachial plexus	7/55 (12.7%)
Lumbosacral plexus	6/55 (10.9%)
Peroneal nerve	6/55 (10.9%)
Ulnar nerve	6/55 (10.9%)
Tibial nerve	5/55 (9.1%)
Median nerve	4/55 (7.3%)
Medial antebrachial cutaneous nerve	3/55 (5.5%)
Sural nerve	2/55 (3.6%)
Vagus nerve	2/55 (3.6%)
Femoral nerve	1/55 (1.8%)
Intercostal nerve	1/55 (1.8%)
Saphenous nerve	1/55 (1.8%)
Digit III	1/55 (1.8%)

Note: Data are presented as *n*/N (%) unless otherwise indicated. Patient-level characteristics refer to 52 patients, whereas lesion-level characteristics refer to 55 histologically confirmed HPNST lesions.

**Table 2:** Histopathological and immunohistochemical characteristics of 55 HPNST lesions. Hybrid neurofibroma/schwannoma was the predominant subtype (92.7%). Ki-67/MIB-1 was available in 74.5% of tumors and was ≤6% in most evaluable cases. S100 expression was retained in all documented cases, CD34 positivity was frequent, and EMA showed capsular positivity in 57.6% of evaluable tumors. Reference neuropathological review was documented in 5 cases. Immunohistochemical assessment was not standardized across centers.

Variable	Value, *n*/N (%)
Histopathological subty pe	
Schwannoma/neurofibroma	51/55 (92.7%)
Schwannoma/perineurioma	2/55 (3.6%)
Triphasic	2/55 (3.6%)
Proliferative index	
Ki-67 category documented	41/55 (74.5%)
Ki-67 <3%	22/41 (53.7%)
Ki-67 4–6%	14/41 (34.1%)
Ki-67 >6%	5/41 (12.2%)
Immunohistochemistry	
S100 expression documented	41/55 (74.5%)
Strong or retained S100 expression	41/41 (100%)
CD34 positivity documented	37/55 (67.3%)
EMA expression documented	33/55 (60.0%)
Capsular EMA positivity	19/33 (57.6%)
EMA negative	14/33 (42.4%)
Neuropathological review	
Reference neuropathological review documented	5/55 (9.1%)

Note: Immunohistochemical staining was performed according to local diagnostic practice and was not standardized across participating centers. Therefore, marker-specific percentages refer to tumors with documented results and should not be interpreted as expression frequencies for the entire cohort. Reference neuropathological review was explicitly documented in five cases; absence of documentation does not necessarily indicate that no additional pathological consultation occurred.

## Data Availability

Data are available from the PNTR Study Group upon reasonable request and in accordance with ethical and legal restrictions.

## Abbreviations

The following abbreviations are used in this manuscript:

CD34	Cluster of differentiation 34
EMA	Epithelial membrane antigen
ERBB2	erb-b2 receptor tyrosine kinase 2
GLUT1	Glucose transporter
HNS	Hybrid neurofibroma/schwannoma
HPNST	Hybrid peripheral nerve sheath tumor
IQR	Interquartile ranges
Ki-67	Ki-67 proliferation marker
MIB-1	MIB-1 antibody
MRI	Magnetic resonance imaging
MPNST	Malignant peripheral nerve sheath tumor
NF1	Neurofibromatosis type 1
NF2	Neurofibromatosis type 2
LE	Lower extremity
PR	Partial resection
PNTR	Peripheral nerve tumor registry
PNST	Peripheral nerve sheath tumor
SWN	Schwannomatosis
SOX10	SRY-box transcription factor 10
UE	Upper extremity
VGLL3	Vestigial-like family member 3
WHO	World Health Organization
